# Expression of the TMPRSS2:ERG fusion gene predicts cancer recurrence after surgery for localised prostate cancer

**DOI:** 10.1038/sj.bjc.6604054

**Published:** 2007-10-30

**Authors:** R K Nam, L Sugar, W Yang, S Srivastava, L H Klotz, L-Y Yang, A Stanimirovic, E Encioiu, M Neill, D A Loblaw, J Trachtenberg, S A Narod, A Seth

**Affiliations:** 1 Division of Urology, Sunnybrook Health Sciences Centre, University of Toronto, Toronto, Canada; 2 Department of Pathology, Sunnybrook Health Sciences Centre, University of Toronto, Toronto, Canada; 3 Laboratory of Molecular Pathology, Molecular and Cellular Biology Research, Sunnybrook Research Institute, University of Toronto, Toronto, Canada; 4 Center for Prostate Disease Research, Uniformed Health Services University of the Health Sciences, Rockville, MD, USA; 5 Department of Radiation Oncology, Sunnybrook Health Sciences Centre, University of Toronto, Toronto, Canada; 6 Division of Urology, University Health Network, University of Toronto, Toronto, Canada; 7 Department of Public Health Sciences, University of Toronto, Toronto, Canada

**Keywords:** prostatic neoplasms, surgery, prognosis, gene fusion

## Abstract

The prostate-specific gene, TMPRSS2 is fused with the gene for the transcription factor ERG in a large proportion of human prostate cancers. The prognostic significance of the presence of the TMPRSS2:ERG gene fusion product remains controversial. We examined prostate cancer specimens from 165 patients who underwent surgery for clinically localised prostate cancer between 1998 and 2006. We tested for the presence of TMPRSS2:ERG gene fusion product, using RT–PCR and direct sequencing. We conducted a survival analysis to determine the prognostic significance of the presence of the TMPRSS2:ERG fusion gene on the risk of prostate cancer recurrence, adjusting for the established prognostic factors. We discovered that the fusion gene was expressed within the prostate cancer cells in 81 of 165 (49.1%) patients. Of the 165 patients, 43 (26.1%) developed prostate-specific antigen (PSA) relapse after a mean follow-up of 28 months. The subgroup of patients with the fusion protein had a significantly higher risk of recurrence (58.4% at 5 years) than did patients who lacked the fusion protein (8.1%, *P*<0.0001). In a multivariable analysis, the presence of gene fusion was the single most important prognostic factor; the adjusted hazard ratio for disease recurrence for patients with the fusion protein was 8.6 (95% CI=3.6–20.6, *P*<0.0001) compared to patients without the fusion protein. Among prostate cancer patients treated with surgery, the expression of TMPRSS2:ERG fusion gene is a strong prognostic factor and is independent of grade, stage and PSA level.

The surgical treatment of localised prostate cancer has been shown to improve prostate cancer-specific mortality (Bill-Axelson *et al*, 2005); however, for many patients with prostate cancer the course is indolent, and in the absence of surgery, many do not experience disease progression to metastasis or death (Albertsen *et al*, 1998). Currently, the established prognostic factors (histologic grade, stage and prostate-specific antigen (PSA) level at diagnosis (Albertsen *et al*, 1998; Barry *et al*, 2001) are insufficient to separate prostate cancer patients who are at high risk for cancer progression from those who are likely to die of another cause. If it were available, a predictive marker would allow physicians to maximise the benefits, and to minimise the side effects, of surgery and of other treatments. To date, molecular markers for prostate cancer progression remain elusive. This is in contrast to the situation for other malignancies, such as breast cancer, where molecular-based prognostic markers have revolutionised treatment (Seidman, 2006).

Potentially useful genetic markers for prostate cancer progression have been identified through gene array analysis. Tomlins *et al* (2005) identified the overexpression of ERG (21q22.3) and ETV1 (7p21.2) in prostate cancer tumour cells after DNA fusion with a prostate-specific gene, TMPRSS2. ERG and ETV1 proteins are members of the ETS family of transcription factors, which are important in several oncogenic pathways (Watson *et al*, 2002; Seth and Watson, 2005). The TMPRSS2 protein is a serine protease that is highly expressed in both normal and cancerous prostate cells, and expression is regulated by androgens (Lin *et al*, 1999; Afar *et al*, 2001). In many cases, the promoter/enhancer region of the TMPRSS2 gene was found to be fused to the coding region of one of the ETS transcription factors, ERG or ETV1. Expression of the fusion gene was found in prostate cancer cell lines, and from prostate cancer tissue obtained from radical prostatectomy specimens.

The potential utility of the TMPRSS2:ERG fusion product as an independent prognostic marker for patients with clinically localised prostate cancer remains controversial. Several studies have compared clinicopathological parameters (Perner *et al*, 2006; Wang *et al*, 2006; Lapointe *et al*, 2007; Rajput *et al*, 2007; Tu *et al*, 2007) and prognostic significance (Wang *et al*, 2006; Attard *et al*, 2007; Demichelis *et al*, 2007; Lapointe *et al*, 2007; Nam *et al*, 2007) of this gene fusion with conflicting results. Two of these studies showed no correlation between histologic grade (Gleason score) (Lapointe *et al*, 2007; Tu *et al*, 2007), while others found positive associations (Attard *et al*, 2007; Rajput *et al*, 2007). Also, some studies have only shown correlation of fusion status with tumour stage (Perner *et al*, 2006; Wang *et al*, 2006). Wang *et al* (2006) examined 119 patients for fusion status from a case–control approach and found significant correlations with tumour stage, but no associations were found with early recurrence. Further, Lapointe *et al* (2007) in another case–control study found no correlations with any clinicopathological parameter and recurrence-free survival.

However, two cohort studies of men with clinically localised prostate cancer who did not undergo treatment (i.e. watchful waiting) showed that men who had TMPRSS2:ERG fusion had lower prostate cancer-specific survival compared to men without fusion expression (Attard *et al*, 2007; Demichelis *et al*, 2007). Patients managed and selected for watchful waiting from these cohorts have different baseline distributions in grade, stage and PSA level to patients treated with surgery and may not be comparable. From a small cohort of 26 patients who underwent surgery for clinically localised disease, we recently showed that those with the fusion expression had a significantly higher rate of recurrence compared to patients who lacked the fusion expression (5-year recurrence rate 79.5 *vs* 37.5%, *P*=0.009) (Nam *et al*, 2007). However, because the follow-up period was short (mean 12 months) and the sample size was small (*n*=26), we could not determine if this effect was independent of other factors, such as grade, stage and PSA level. We also examined the significance of ETV1 fusion, but did not find any association with cancer recurrence (Nam *et al*, 2007).

Thus, it remains unclear whether the TMPRSS2:ERG gene fusion is only a surrogate marker for established prognostic factors of grade and stage, or whether it is an independent molecular-based marker for disease recurrence with no association with grade or stage, particularly for patients who are candidates for surgery for clinically localised prostate cancer. To examine further these associations, we sought the presence of TMPRSS2:ERG gene fusion in 165 frozen prostate cancer samples from patients who underwent radical prostatectomy for localised prostate cancer. We compared the frequencies of TMPRSS2:ERG gene fusion status between the clinicopathological variables of grade, stage and PSA level. We also compared the rates of disease recurrence between patients with and without the TMPRSS2:ERG gene fusion expression.

## MATERIALS AND METHODS

### Study subjects

Patients were drawn from a series of 300 men who underwent radical prostatectomy as the sole treatment for clinically localised prostate cancer, between 1998 and 2006, at Sunnybrook Health Sciences Center (Toronto, Canada). Since 1998 and with the approval of the research ethics board, a prostate tumour tissue bank was established. Prostate specimens were sampled at surgery and immediately frozen. Patients were excluded for this study if they used androgen deprivation therapy or chemotherapy before surgery (*n*=3) or if they were treated with adjuvant radiation or androgen deprivation therapy (*n*=21). For all patients the serum PSA fell to an undetectable level (<0.02 ng ml^−1^) following surgery. Of the remaining 276 patients, 111 patients had cancer cells that were not identified in the banked samples obtained from the prostatectomy or insufficient tissue was available for RNA extraction and RT–PCR analysis, leaving 165 patients available for the follow-up analysis.

### Prostate sample collection

All samples were taken from the prostate at the time of prostatectomy. A section from the mid-portion of the prostate was snap-frozen in liquid nitrogen and stored at −80°C. The majority of prostate tumours were not palpable within the prostatectomy specimen, and therefore, the samples obtained from the prostate were considered to be random. The banked slices of specimens were photocopied, oriented (anterior, posterior, right and left), quadrisected and cut in 5 *μ*m sections on the cryostat. The sections were stained with H&E and then reviewed by the pathologist (LS). The areas of tumour were marked on the stained slides and on the photocopied diagram. The marked areas were used to extract the tissue for total RNA extraction.

Total RNA was extracted from the frozen prostate cancer tissue by homogenising in Trizol (Invitrogen Corporation, Carlsbad, CA, USA) followed by ethanol precipitation. The RNA pellet was dissolved in RNase-free H_2_O and quality was checked using 2100 Bioanalyzer (Agilent Technologies, Inc., Santa Clara, CA, USA). All tumour samples were obtained from the primary Gleason score grade. For example, for a Gleason Score 7 (4+3) tumour, samples were obtained from the Gleason Grade 4 pattern.

### RT–PCR and direct DNA sequencing

**Figure 1:**
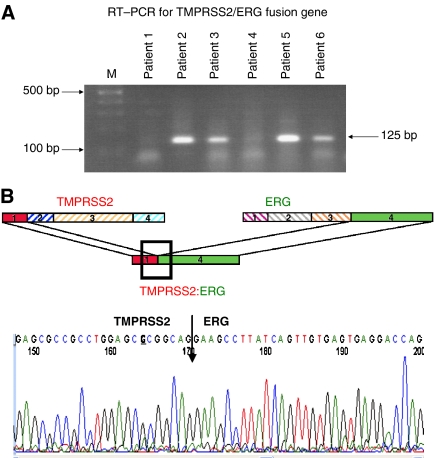
(**A**) DNA in agarose gel after TMPRSS2:ERG-specific RT–PCR of RNA from primary prostate cancer tissue. Shown are PrR amplified products, from a few representative cases, resolved by electrophoresis on a 2% agarose gels. Primer sets producing an expected size of 125 bp poducts are shown. (**B**) Schematic diagram and electropherograms of TMPRSS2:ERG-specific RT–PCR amplified products. Diagramed are TMPRSS2 and ERG variant 2 (Genbank NM_00449) gene exons and fusion transcripts, with deleted exons in hatched colour, retained exons in solid colour, and the fusion junction boxed. Shown is a fusion junction for one of the cases, after electrophoresed DNA in bands (Figure 1A) was excised, purified and sequenced using an ABI 3700 (Applied Biosystems) automated DNA sequencer.

The presence of the TMPRSS2:ERG fusion gene was established using RT–PCR for all 165 prostate cancer patient samples, described elsewhere (Nam *et al*, 2007). Briefly, 1 μg of total RNA was reverse transcribed into cDNA using QuantiTect Reverse Transcription Kit (Qiagen Inc., Valencia, CA, USA) in the presence of random and oligo-dT primers. All reactions were performed with both the forward and reverse primer sets (F-TAGGCGCGAGCTAAGCAGGAG, R-GTAGGCACACTCAAACAACGACTGG) that yields a 125-bp and (F-CAGGAGGCGGAGGCGGA, R-GGCGGTTGTAGCTGGGGGTGAG) that yields a 595-bp product as described by Tomlins *et al* (2005). PCR amplified products were resolved by electrophoresis on a 2% agarose gels and bands were excised, purified and sequenced using the ABI 3700 (Applied Biosystems, Foster City, CA, USA) automated DNA sequencer (Figure 1) (Nam *et al*, 2007).

### Patient follow-up

Clinical data and follow-up informations were collected prospectively. The medical records were systematically reviewed using standardised data entry forms by trained data abstractors and stored within a prostate cancer-specific database. Clinical follow-up consisted of four assessments in the year following surgery, two assessments in the second year and one assessment every year thereafter. At each follow-up, patients had a clinical evaluation, and a PSA test.

### Data analysis

To determine whether or not TMPRSS2:ERG gene fusion was a prognostic factor for cancer recurrence, we conducted a survival analysis, from date of diagnosis to date of first biochemical recurrence Biochemical recurrence was defined as a PSA increase of at least 0.2 ng ml^−1^ on at least two separate consecutive measurements that are at least 3 months apart. The date of recurrence was coded as the date of the first PSA recording of ⩾0.2 ng ml^−1^. Patients were considered to be at risk from the date of surgery until recurrence or until the date of the last PSA test. Patients that were lost to follow-up were censored at the date of their last PSA test. Within the study period, only one patient died (cause unrelated to prostate cancer). He was censored at the last PSA test.

Rates of (biochemical) recurrence were compared between patients with tumours that expressed TMPRSS2:ERG gene fusion and with those who lacked the fusion product, using the Kaplan–Meier method. The effect of TMPRSS2:ERG gene fusion was then examined in a multivariable Cox proportional hazard model, adjusting for age, grade, stage and PSA level at diagnosis. Pathologic stage was categorised into three groups: (1) organ confined; (2) extracapsular extension and (3) seminal vesicle involvement (Nam *et al*, 2003). Histologic grade was categorised into three groups: (1) Gleason Score 5 and 6; (2) Gleason Score 7 and (3) Gleason Score 8–10. Prostate-specific antigen level at diagnosis was treated as a continuous variable. All data analysis was conducted using the SAS System V9.1 (Carey, NC, USA).

## RESULTS

### Patient demographics and recurrence rates

The mean age at diagnosis of the 165 patients was 61.8 years (s.d.=7.4 years, range=31–75 years). The distributions of pathologic stage, margin status and PSA are described in Table 1. Among the cohort, 44.1% of patients had cancers confined to the prostate gland and the majority of cancers were of Gleason Score 7 (Table 1). The mean follow-up time was 28.4 months (range=3–96 months). The overall median follow-up was 20 months. The median follow-up in the fusion negative arm was 27 months and in the fusion positive arm was 13 months. Of the 165 patients, 43 (26.1%) developed a biochemical recurrence, defined as a PSA level of >0.02 ng l^−1^ on at least two separate consecutive measurements that are at least 3 months apart. The crude 5-year biochemical recurrence rate for the entire cohort was 33.8% (95% CI=25.0–44.8%).

### Prognostic significance of TMPRSS2:ERG gene fusion

**Figure 2:**
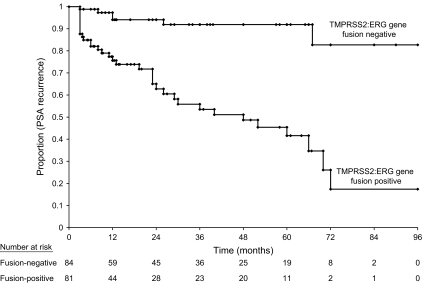
Kaplan–Meier survival estimates for patients with and without TMPSSR2:ERG gene fusion using biochemical recurrence as the primary end point.

The presence of TMPRSS2 fusion with ERG in prostate cancer samples was examined by RT–PCR of RNA for all cases. Prostate samples from 81 of 165 (49.1%) patients were found to be positive for fusion transcripts for the TMPRSS2:ERG fusion gene (Figure 1). All fusion transcripts positive samples produced the expected 125 or 595 bp bands depending upon which primer set used. DNA sequencing indicated that most correspond to the fusion of TMPRSS2 Exon 1 with Exon 4 of ERG transcript variant 2 (Genbank NM_00449). This fusion mRNA was reported to be the most frequently observed fusion of the two genes, for it was found in >90% of all tumours tested (Wang *et al*, 2006; Nam *et al*, 2007). Among the 81 patients who had TMPRSS2:ERG fusion transcripts within their tumour tissue, 37 (45.7%) experienced biochemical recurrence, whereas among the 84 patients who had tumours that lacked fusion, only six (7.1%) experienced a relapse (odds ratio=10.9, 95% CI=4.3–27.9, *P*=10^−7^). On the basis of the Kaplan–Meier survival estimates, the group of patients who had tumours with TMPRSS2:ERG gene fusion had a much greater rate of recurrence at 5 years (58.4%) than patients who lacked the fusion gene (8.1%; *P*<0.0001) (Figure 2).

In the univariable analysis, TMPRSS2:ERG fusion was a strong predictor for disease recurrence (hazard ratio=8.2, 95% CI=3.4–19.5, *P*<0.0001) (Table 2). The established prognostic factors of histologic grade, pathologic stage and PSA level at diagnosis were also significant predictors for disease recurrence (Table 2). After adjustment for these factors, the prognostic importance of the fusion protein was not diminished (hazard ratio=8.6; 95% CI=3.6–20.6, *P*<0.0001) (Table 2). Among the prognostic factors, only grade and fusion status were significant predictors for disease recurrence. Of the two, fusion gene status had a stronger effect on disease recurrence than did each incremental increase in grade category (hazard ratios=8.5 and =6.3, respectively).

To determine whether or not the presence of the TMPRSS2:ERG fusion correlated with other prognostic factors, we compared the proportions of patients with the gene fusion in patient subgroups defined by grade, stage and PSA level. The proportions were similar in each grade and stage category, and the distribution of PSA levels did not significantly differ between patients with or without fusion (Table 1). Also, of the six patients who recurred without any evidence of fusion, five of the six patients had a Gleason Score of 7 or more.

We examined further whether TMPRSS2:ERG gene fusion was predictive of recurrence stratified by grade and stage. The 5-year recurrence-free survival for patients with Gleason Score 5–6, 7 and 8–10 were 76.1 (95% CI=53.4–88.7%), 64.0% (95% CI=50.1–74.9%) and 48.5% (95% CI=8.8–80.6%), respectively. For all grade categories, patients with TMPRSS2:ERG gene fusion was associated with increased risk for recurrence (Table 3). Similarly, patients with gene fusion had a higher risk for recurrence for all stage categories (Table 3).

## DISCUSSION

We have identified an important genetic-based prognostic factor for prostate cancer recurrence among patients who are treated for clinically localised cancer with surgery alone. Since Tomlins *et al* (2005) first described the association between TMPRSS2:ERG gene fusion expression and prostate cancer, several other studies have addressed the potential clinical implications of the discovery. All case–control studies (Perner *et al*, 2006; Wang *et al*, 2006) did not show associations with disease recurrence, but did show correlations with stage or grade. Perner *et al* (2006) studied 118 prostate cancer samples from various prostate cancer groups at different stages using FISH and found 60.3% of patients with fusion and a significant correlation was found with stage at diagnosis. Wang *et al* (2006) identified the presence of fusion using RT–PCR in 59% of patients, but no correlation with prostate cancer recurrence was identified. However, in their study, cases and controls were not derived from the same source. From a case–control study by Lapointe *et al* (2007), where 54 patients with localised cancer and 9 patients with metastasis were examined for fusion status, no correlations were found with stage, grade or recurrence. On the other hand, large cohort studies of patients managed with watchful waiting did show correlations with a positive fusion status being associated with reduced survival (Attard *et al*, 2007; Demichelis *et al*, 2007).

In the current study, neither grade, stage nor PSA level correlated with the presence of TMPRSS2:ERG gene fusion. This could be due to insufficient power to examine for these multiple comparisons. However, the distribution of gene fusion expression by grade, stage and PSA are highly similar between the fusion positive and negative patients, and the Kaplan–Meier recurrence rates were different by fusion expression for each category of grade and stage (Table 3). Further, Mosquera *et al* (2007) showed from morphological analysis of tumour tissue that gene fusion expression was correlated to other histologic features, and not related to Gleason grading or pathologic staging. This suggests a unique biologic mechanism that requires further elucidation. Our results would be consistent with these findings.

In the multivariable analysis, gene fusion was the strongest predictor for disease recurrence followed by grade. We could not examine for potential statistical interaction due to our sample size, which could be responsible for the changes in value of the hazard ratios in the multivariable analysis. Before this study, tumour grade was considered to be the most important prognostic factor (Barry *et al*, 2001). Within our model, grade was still an important factor, but the relative risk for recurrence was more marked for TMPRSS2:ERG gene fusion status than for grade. Indeed, of the six patients who recurred without fusion, five of them had a Gleason 7 or more tumours, that has a higher risk of relapse than Gleason Score 6 tumours.

A limitation to our study is the use of PSA recurrence as the primary end point. It would be ideal to use prostate cancer-specific mortality. Prostate-specific antigen recurrence (>0.2 ng ml^−1^) has been shown to be surrogate marker for prostate cancer-specific mortality from large, independent cohorts (Walsh *et al*, 1994; Zincke *et al*, 1994; Pound *et al*, 1999; D'Amico *et al*, 2003). Pound *et al* (1999) showed that patients initially described by Walsh (1994) who develop a PSA recurrence after surgery develop metastasis over a median time of 8 years. Zincke *et al* (1994) compared PSA recurrence rates, cause-specific mortality and overall survival among 3170 patients treated with surgery and found strong and consistent correlations between these outcome variables and grade, stage and PSA level. D'Amico *et al* (2003) further showed that higher rates of PSA rise after surgery or radiation was associated with earlier prostate cancer-specific death. Because we used PSA recurrence as our end point, patients who received neoadjuvant or adjuvant treatments were excluded given that the time to PSA recurrence may be biased, introducing potential selection bias. A larger cohort with longer follow-up for prostate cancer mortality will be needed to closely examine these relationships with gene fusion expression.

Another limitation was that the majority of our patients had Gleason Score 7 cancer and few patients had tumours of Gleason score 6 or less. This is likely due to tumour sampling at the time of prostatectomy; patients with Gleason Score 6 or less in general have a lower volume of cancer and the probability of sampling the cancer would therefore be lower than for patients with cancers of Gleason 7 or more. This resulted in a large number of patients being excluded which is likely responsible for the high rate of recurrence found from this study. Current methods in examining for expression of gene fusion are limited to frozen tissue, which is susceptible to tumour sampling error. Future studies should be able to overcome this limitation, such as analysing RNA from paraffin-embedded samples or immunohistochemistry. Nevertheless, the prognostic significance of grade, stage and PSA level in the current study are similar to the findings of other larger, contemporary series in North America (Barry *et al*, 2001).

Many other rare transcripts of this gene fusion have been described (Soller *et al*, 2006; Wang *et al*, 2006; Clark *et al*, 2007; Liu *et al*, 2007; Tu *et al*, 2007). We only examined the most common fusions of TMPRSS2 exon 1 with exon 4 of ERG transcript. Alternative fusions are possible, but having two or more distinct translocations on each allele in a single tumour area is not likely rather, these transcripts may arise from alternative splicing of the fused exons, after transcription. Alternative splicing of ERG gene is well studied, with up to 17 variants being described (Soller *et al*, 2006; Clark *et al*, 2007). Several studies have consistently confirmed that fusion at exon 1 of TMPRSS2 is the most common variant (Tu *et al*, 1996; Wang *et al*, 2006; Clark *et al*, 2007). However, Clark *et al* (2007) also found that distinct patterns of hybrid transcripts from separate areas of the same tumour which would make associations of clinical significance more difficult. These and other separate fusion transcripts will require further study to examine for their association with prostate cancer prognosis.

If these findings are confirmed, they might have an important impact on the treatment of clinically localised prostate cancer. In a randomised trial, Bill-Axelson *et al* (2005) showed that patients who had prostatectomy had a better cancer-specific survival than did patients who did not undergo surgery for clinically-localised prostate cancer. However, the gains in life expectancy and survival associated with surgery were small. Using TMPRSS2:ERG gene fusion status to differentiate between patients who are at high risk for disease progression may eventually help to identify patients who would benefit the most from treatment.

## Figures and Tables

**Table 1:** Distribution of baseline factors of all patients and correlation between TMPRSS2:ERG gene fusion, histologic grade, pathologic stage and PSA level at diagnosis

		**TMPRSS2:ERG gene fusion expression**		
**Prognostic factor**	**All patients (*n*=165)**	**Positive (*n*=81)**	**Negative (*n*=84)**	***P*-value**
*Histologic grade*				
Gleason Score 5–6	41	21 (51.2%)	20 (48.7%)	0.67
Gleason Score 7	113	56 (49.6%)	57 (50.4%)	
Gleason Score 8–10	11	4 (36.3%)	7 (63.6%)	
				
*Pathologic stage*				
Organ confined	74	35 (47.3%)	39 (52.7%)	0.92
Extracapsular extension	79	40 (50.6%)	39 (49.4%)	
Seminal vesicle involvement	12	6 (50%)	6 (50%)	
				
*PSA level at diagnosis (ng ml*^−*1*^)				
Mean (s.d.)	9.3 (7.3)	9.5 (5.7)	9.1 (8.6)	0.73
Median	7.3	8.0	7.0	0.14
Range	2.1–64	3.0–38.9	2.1–64	

**Table 2:** Univariable and multivariable Cox regression analysis of pathologic stage, margin status, PSA level at diagnosis and TMPRSS2:ERG gene fusion

**Covariate**	**Crude hazard ratio**	**95% CI**	***P*-value**	**Adjusted hazard ratio**	**95% CI**	***P*-value**
*Histologic grade*						
Gleason Score⩽6	1.0			1.0		
Gleason Score 7	1.9^a^	0.9–4.2	0.11	1.8^a^	0.8–3.9	0.17
Gleason Score 8–0	4.6^c^	1.3–16.0	0.02	6.3^a^	1.7–23.6	0.006
						
*Pathologic stage*						
Organ confined	1.0			1.0		
Extracapsular extension	1.9	0.9–4.2	0.11	1.1	0.6–2.2	0.77
Seminal vesicle Involvement	4.6	1.3–16.0	0.01	1.4	0.5–4.4	0.54
						
PSA	1.04^b^	1.0–1.1	0.01	1.02	0.9–1.1	0.30
						
*TMPRSS2:ERG Gene fusion expression*						
Negative	1.0			1.0		
Positive	8.2	3.4–19.5	<0.0001^c^	8.6	3.6–20.6	<0.0001^c^

a Test for trend, *χ*^2^=5.4, *P*=0.02 for crude; and *χ*^2^=5.6, *P*=0.02 for adjusted value.

b Hazard ratio based on per unit (ng ml^−1^) increase in PSA level.

c *χ*^2^ (1 d.f.)=22.5 for crude and 22.6 for adjusted hazard ratio.

**Table 3:** Five-year Kaplan–Meier (KM) biochemical survival estimates by TMPRSS2:ERG gene fusion status stratified by histologic grade and pathologic stage

	**TMPRSS2:ERG fusion status**				
	**Positive**		**Negative**		
**Prognostic factor**	**Number of Events**	**5-year KM biochemical survival rate (median follow-up months)**	**Number of events**	**5-year KM biochemical survival rate (median follow-up months)**	*P*-value*
*Grade*					
Gleason Score 5–6	7/21 (33.3%)	60.6% (20)	1/20 (5.0%)	93.3% (24)	0.02
Gleason Score 7	27/56 (48.2%)	37.6% (12)	4/57 (7.0%)	92.5% (29)	<0.0001
Gleason Score 8–10	3/4 (75.0%)	0.0% (5)	1/7 (14.3%)	85.7% (10)	0.07
					
*Stage*					
Organ confined	12/35 (34.3%)	59.0% (20)	4/39 (10.3%)	85.7% (26)	0.007
Extraprostatic extension	21/40 (52.5%)	27.0% (12)	1/39 (2.6%)	100.0% (34)	<0.0001
Seminal vesicle involvement	4/6 (66.7%)	0.0% (6)	12/35 (34.3%)	83.3% (7)	0.13

* *P*-value based on Kaplan–Meier log-rank test for significance.
